# Myocardial extracellular volume quantification with computed tomography—current status and future outlook

**DOI:** 10.1186/s13244-023-01506-6

**Published:** 2023-09-25

**Authors:** Giulia Cundari, Nicola Galea, Victor Mergen, Hatem Alkadhi, Matthias Eberhard

**Affiliations:** 1 Diagnostic and Interventional Radiology, University Hospital Zurich, University of Zurich, Raemistrasse 100, 8091 Zurich, Switzerland; 2 Department of Radiological, Oncological and Pathological Sciences, Sapienza University of Rome, Rome, Italy; 3 Radiology, Spital Interlaken, Spitäler FMI AG, Unterseen, Switzerland

**Keywords:** Computed tomography, Extracellular volume, Late enhancement, Myocardial fibrosis, Tissue characterization

## Abstract

Non-invasive quantification of the extracellular volume (ECV) is a method for the evaluation of focal and diffuse myocardial fibrosis, potentially obviating the need for invasive endomyocardial biopsy. While ECV quantification with cardiac magnetic resonance imaging (ECV_MRI_) is already an established method, ECV quantification with CT (ECV_CT_) is an attractive alternative to ECV_MRI_, similarly using the properties of extracellular contrast media for ECV calculation. In contrast to ECV_MRI_, ECV_CT_ provides a more widely available, cheaper and faster tool for ECV quantification and allows for ECV calculation also in patients with contraindications for MRI. Many studies have already shown a high correlation between ECV_CT_ and ECV_MRI_ and accumulating evidence suggests a prognostic value of ECV_CT_ quantification in various cardiovascular diseases. Adding a late enhancement scan (for dual energy acquisitions) or a non-enhanced and late enhancement scan (for single-energy acquisitions) to a conventional coronary CT angiography scan improves risk stratification, requiring only minor adaptations of the contrast media and data acquisition protocols and adding only little radiation dose to the entire scan.

**Critical relevance statement**

This article summarizes the technical principles of myocardial extracellular volume (ECV) quantification with CT, reviews the literature comparing ECV_CT_ with ECV_MRI_ and histopathology, and reviews the prognostic value of myocardial ECV quantification for various cardiovascular disease.

**Key points**

• Non-invasive quantification of myocardial fibrosis can be performed with CT.

• Myocardial ECV quantification with CT is an alternative in patients non-eligible for MRI.

• Myocardial ECV quantification with CT strongly correlates with ECV quantification using MRI.

• Myocardial ECV quantification provides incremental prognostic information for various pathologies affecting the heart (e.g., cardiac amyloidosis).

**Graphical Abstract:**

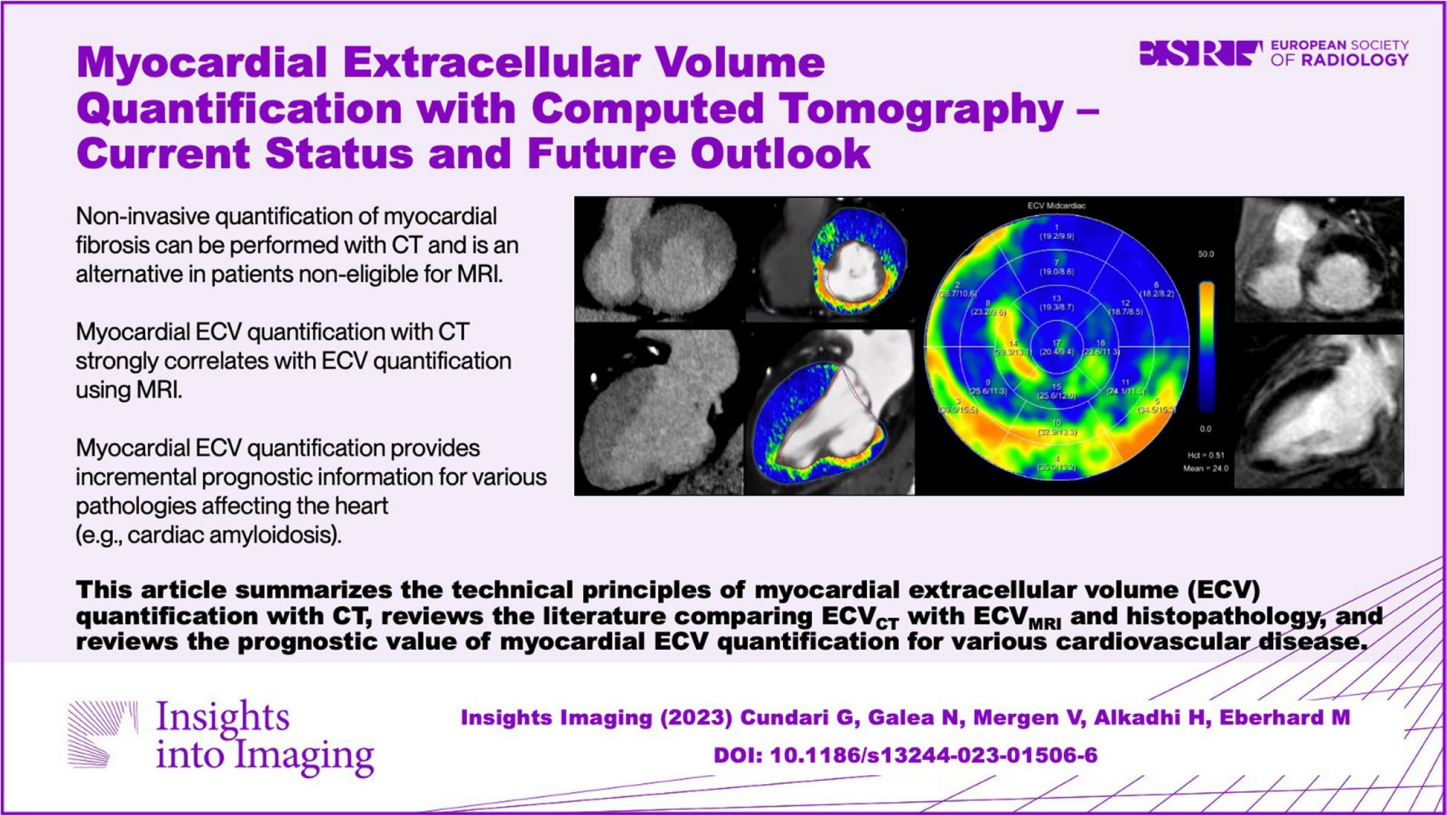

## Introduction

Myocardial fibrosis is a common endpoint of several diseases affecting the myocardium [1]. Focal replacement fibrosis after myocardial infarction or diffuse interstitial fibrosis with accumulation of collagen in the extracellular space as a consequence of non-ischemic or infiltrative cardiomyopathies increase the myocardial stiffness and hamper myocardial contractility [2–7]. The reference standard for quantification of myocardial fibrosis is invasive endomyocardial biopsy with its inherent procedural risks and the possibility of sampling errors as only a small portion of the myocardium can be evaluated [8]. Therefore, non-invasive quantification of the myocardial ECV gained increasing interest in the past decade. It enables the global assessment of myocardial fibrosis and thus may represent a non-invasive quantitative measure for risk prediction and prognostication in patients with various cardiac diseases [9, 10].

While non-invasive quantification of the extracellular volume (ECV) with cardiac magnetic resonance imaging (ECV_MRI_) has already become a clinically established, quantitative marker [11], recently, ECV quantification with CT (ECV_CT_) evolved as a robust and reliable alternative. ECV_CT_ represents a quantitative means for the evaluation of myocardial fibrosis on a cardiac late enhancement scan. In comparison to MRI, CT is more widely available, cheaper and faster and enables to obtain submillimeter isotropic volumetric image data. However, CT has the drawback of ionizing radiation, the need for potentially nephrotoxic contrast media (CM), and the lower soft tissue contrast resolution compared to MRI [12].

## Extracellular distribution of iodinated contrast media

Non-invasive ECV measurements with CT or MRI depend on the pharmacokinetics of extracellular CM. In this regard, gadolinium and iodine-based CM agents share similar properties. After injection, the CM agents diffuse passively from the vascular space into the extracellular space down a concentration gradient. Later, the concentration gradient reverses and the CM agents reenter the vascular space. At a certain (*late*) timepoint after CM injection, a state of “quasi” equilibrium is reached, where tissue contrast enhancement parallels that of the blood. After reaching an equilibrium, both the intravascular and extracellular CM concentration declines with time due to the elimination of CM by the kidneys [12].

**Fig. 1:**
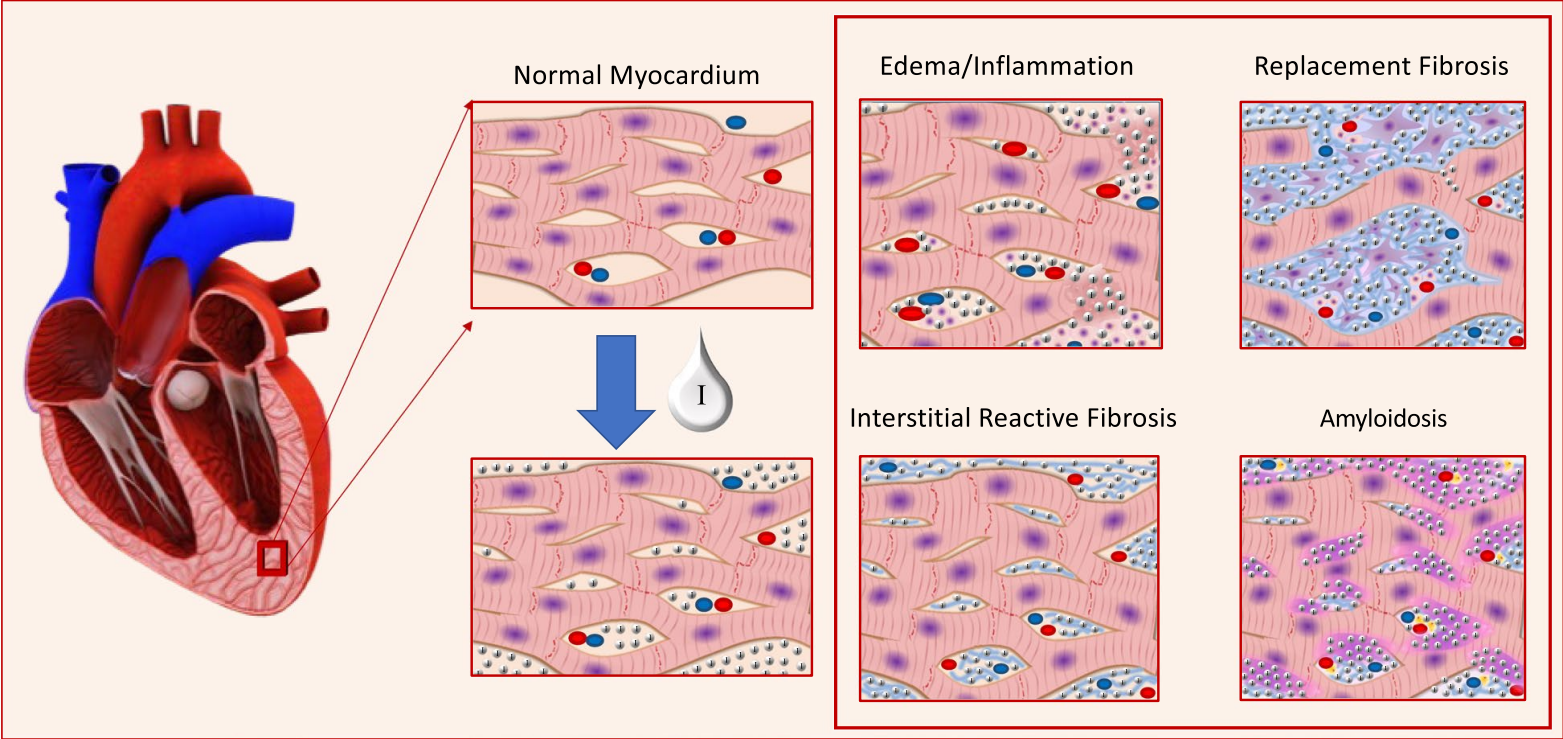
Schematic illustration of myocardial fibrosis. Left: normal myocardium and iodine distribution after contrast media administration. Right: four pathologic conditions related to the expansion of the extracellular matrix: inflammation with edema; replacement fibrosis; interstitial reactive fibrosis and amyloidosis

In myocardial tissue with an expansion of the extracellular matrix (ECM) due to an expansion of the collagen volume fraction, edema or amyloid deposition, the ratio between the amount of extracellular CM in the myocardium and the amount of CM in the vascular space is higher compared to healthy myocardium. The properties of both gadolinium and iodine are used to quantify these differences via measuring the T1-relaxation by MRI or the CT attenuation, both being proportional to the concentration of the respective CM agent. A schematic illustration about the extracellular space is provided in Fig. 1.

While both (MRI and CT) techniques for the non-invasive quantification for ECV emerged around the same time [13, 14], initially, ECV_MRI_ gained more attraction due to the widespread use of late gadolinium enhancement imaging to assess focal myocardial fibrosis. In these cases, ECV_MRI_ added the possibility to also assess global or diffuse myocardial fibrosis. Initially, CT was characterized by only small HU differences between normal and pathologic myocardium [12] hindering widespread clinical use. The recent introduction of low tube voltage scanning and dual-energy techniques increased the contrast-to-noise ratio of late enhancement scans improving the delineation of pathologic myocardium and thus triggered the recent interest in late enhancement scanning and ECV quantification with CT.

## Non-invasive quantification of ECVCT in comparison to ECVMRI

ECV evaluation with cardiac MRI is a well-established method to non-invasively assess the presence of interstitial myocardial fibrosis. Despite the standard reference role of MRI, the technique is characterized by several limitations including the relatively long acquisition time, the requirement of T1 mapping techniques, and the necessity for expertise in the acquisition and analysis of the images being susceptible to artifacts. In this regard, CT offers the opportunity to assess ECV in a shorter acquisition time being less susceptible for artifacts. Moreover, the access to CT scanners being capable of ECV assessment is easier compared to MRI, and CT is a cheaper and faster method to assess ECV in a non-invasive way. Furthermore, CT allows to perform ECV evaluations in patients with metal implants or devices potentially reducing image quality and interpretability of cardiac MRI, in patients with contraindications to MRI, and in patients with claustrophobia [12, 15]. In contrast to cardiac MRI, CT provides a volumetric 3-dimensional ECV assessment with high-resolution data and isotropic voxels.

The disadvantages of CT are the radiation dose, the potential nephrotoxicity from CM administration in patients at risk for contrast-induced nephropathy [16], and the limited contrast-to-noise ratio, as detailed below.

## How is ECV calculated?

The calculation of ECV is based on the ratio between the concentration of CM in the myocardium and in the plasma part of the blood pool, therefore measuring the hematocrit (Ht) of the patient is required to convert whole blood to plasma concentrations.

For single-energy CT, the acquisition of two scans is mandatory to calculate ECV_CT_: an ECG-gated non-enhanced CT scan and an ECG-gated late enhancement scan in the equilibrium phase, acquired between 3 and 10 min after CM administration as detailed below. The differences of Hounsfield units (∆HU) between the pre- and the post-contrast scans are used to assess the distribution of CM, using the following formula [17]:

$$\mathrm{ECV}=\left(1-\mathrm{Ht}\right)\times\left({\Delta\mathrm{HU}}_{\mathrm{myocardium}}/({\Delta\mathrm{HU}}_{\mathrm{bloodpool}}\right)$$

Measurements of HU are obtained by drawing a region of interest (ROI) in the left ventricular cavity for blood pool values and in the myocardium to assess the amount of extracellular CM in the myocardium [14, 18].

However, this single-energy CT method can be limited by misregistration issues occurring during manual tracing of the ROIs (especially in the non-enhanced scan, in which the visualization of myocardial septal wall can be difficult) [19], due to an automatic voxel-wise misregistration or in case of variable heart rates (non-matching acquisition between non- and late-enhancement scans) [18].

In this scenario, the use of dual energy-capable CT scanners has improved the robustness of this technique. Spectral separation of late enhancement scans acquired with different tube voltages or with energy-sensitive detectors allows for material decomposition and, thus, quantification of iodine [15].

With this method, the quantification of ∆HU can be directly performed on iodine maps obtained from late enhancement scans without the need for a non-enhanced acquisition [17], thus making the ECV_CT_ calculation easier compared to the subtraction method. The formula used for ECV_CT_ in case of spectral imaging acquisition is:

$$\mathrm{ECV}=\left(1-\mathrm{Ht}\right)\times\left({\mathrm{Iodine}}_{\mathrm{Myocardium}}/{\mathrm{Iodine}}_{\mathrm{Bloodpool}}\right).$$

**Fig. 2:**
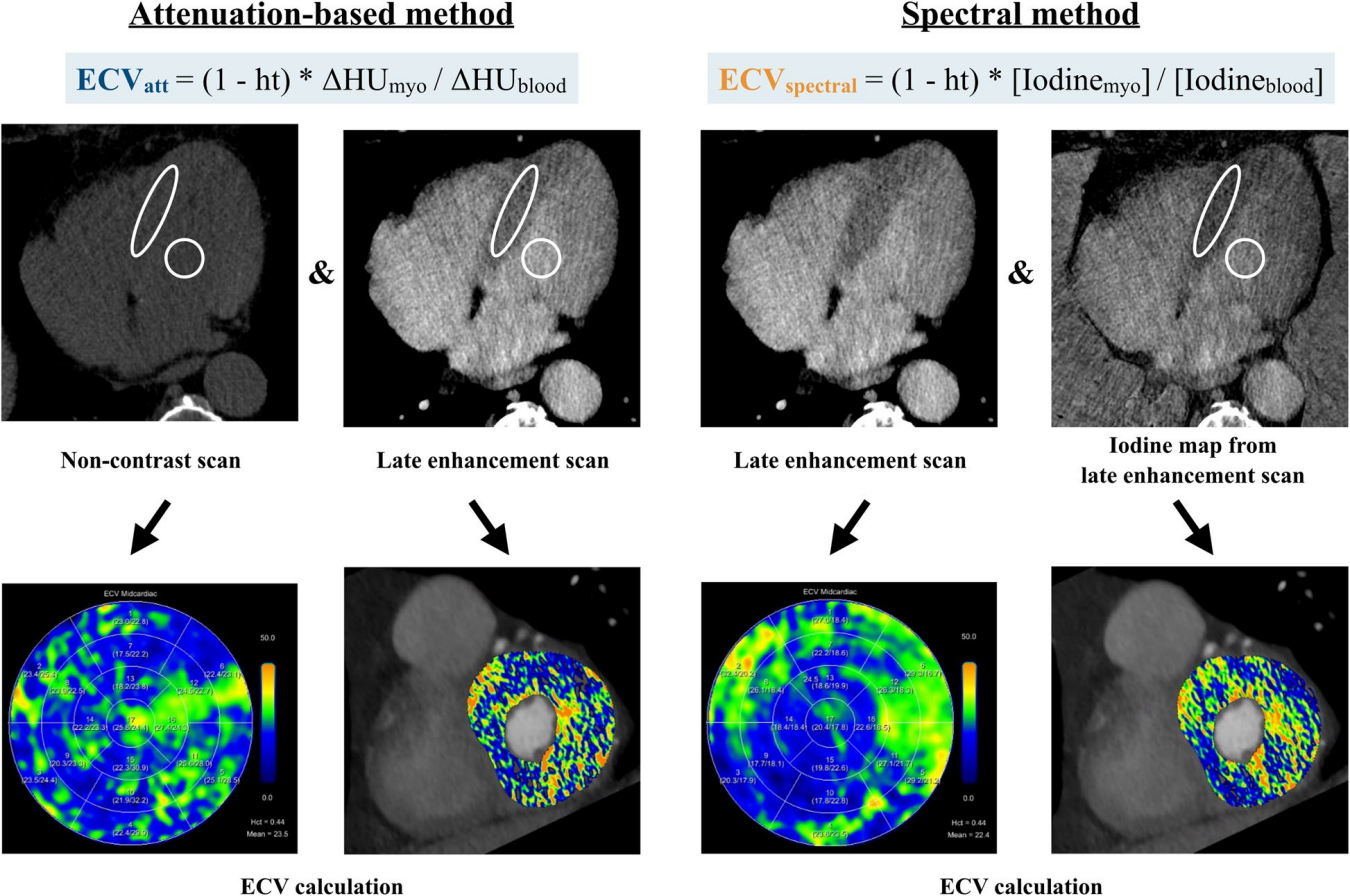
Extracellular volume (ECV) calculation using the single-energy attenuation-based and the spectral method in a 77-year-old male patient. ECV was not elevated with a mean ECV of 23.5% and 22.4% using the attenuation-based and spectral method, respectively. No focal scar was seen, which was confirmed by previous CMR. Abbreviations: Ht, hematocrit; ΔHU_myo_, change in attenuation of the myocardium; ΔHU_blood_, change in attenuation of the blood pool; [Iodine_myo_], iodine concentration within the myocardium; [Iodine_blood_], iodine concentration within the blood pool

A schematic illustration showing ECV calculation with CT is shown in Fig. 2.

## ECV quantification with CT—acquisition protocols

### Late enhancement scan timing

Several acquisition protocols for late enhancement CT scans in the equilibrium phase of iodinated CM distribution were evaluated in recent years using different CM administration protocols with various time delays for the late phase acquisition. Table 1 shows a summary of studies evaluating ECV_CT_ quantification, detailing scan, reconstruction, and contrast media protocols.

**Table 1:** Summary of studies evaluating ECV quantification with CT, detailing scan, reconstruction, and contrast media protocols

	**Scanner type**	**Non-enhanced scan**	**Timing of LE scan [min]**	**Slice thickness/**	**Tube voltage**	**ECG-gating**	**Contrast media [mgI/mL]**	**Dose of contrast media**	**Effective radiation dose**^**a**^
**Single-energy protocols**									
Nacif, 2012 [14]	Aquilion One (Toshiba)	Yes	10	3 mm	120 kVp	Prospective	Iopamidol (370)	Bolus of 125 mL ± 24 mL	1.98 ± 0.16 mSv for non-enhanced and LE scan
Bandula, 2013 [12]	Somatom Sensation 64 (Siemens)	Yes	25	5 mm	120 kVp	Retrospective	Iohexol (300)	Bolus of 1 mL/kg body weight + infusion of 1.88 mL/kg/h	10.7 ± 3.4 mSv (total cardiac CT protocol)
Hamdy, 2018 [20]	Somatom Force (Siemens)	Yes	3, 5, 7	1 mm	80 kVp	Prospective	Iopamiron (370)	Bolus of 1.6 mL/kg body weight	11.8 ± 2.9 mSv (total cardiac CT protocol)
Scully, 2020 [21]	Somatom Force (Siemens)	Yes	3, 5	2 mm	80 kVp	Prospective	Iohexol (300)	Bolus of 90 mL	5.1 ± 0.3 mSv for non-enhanced and LE scan
Tamarappoo, 2020 [10]	Somatom Definition Flash (Siemens)	Yes	5	3 mm	120 kVp	Prospective	Iohexol (350)	Bolus of 100 mL	25.7 ± 6.9 mSv (total cardiac CT protocol)
**Dual-energy protocols**									
Lee, 2016 [18]	Somatom Definition Flash (Siemens)	No	12	0.75 mm	100/140 kVp	Retrospective	Iopamidol (370)	Bolus of 1.8 mL/kg body weight	5.59 ± 0.85 mSv (total cardiac CT protocol)
Abadia, 2019 [15]	Somatom Force (Siemens Healthcare)	No	7	1.5 mm	90/150 kVp	Prospective	Iopamidol (370)	Bolus of 65 mL	3.61 mSv [2.78–4.21] (total cardiac CT protocol)
Oda, 2019 [22]	iQon Spectral CT (Philips)	No	7	0.67 mm	120 kVp	Prospective	Iopamidol (370)	Bolus of 1.5 mL/kg body weight	4.8 ± 1.6 mSv for LE scan
Ohta, 2020 [23]	Discover CT 750 HD (GE)	No	7–8	0.625 mm	80/140 kVp	Prospective	Iopamidol (370)	Bolus of 0.9 mL/kg body weight + infusion until 1.4 mL/kg/scan	3.42 ± 0.87 mSv for LE scan
Dubourg, 2021 [17]	Discover CT 750HD (G)	No	7	0.625 mm	80/140 kVp	Prospective	Iohexol (350)	Bolus of 65 mL	1.89 ± 0.38 mSv for LE scan
Qi, 2022 [24]	Somatom Force (Siemens)	No	7	0.6 mm	90/150 kVp	Prospective	Iopromide (370)	Two boli of 50 mL + 50 mL	5.9 mSv [4.5–8.4] (total cardiac CT protocol)
**Photon-counting detector CT scanner**									
Mergen, 2022 [25]	NAEOTOM Alpha (Siemens)	Yes	5	1.5 mm	120 kVp	Prospective	Iopromide (370)	Bolus of 100 mL	1.2 mSv [0.97–1.75] for LE scan

*LE* Late enhancement

^a^Effective radiation dose was calculated by multiplying the DLP by a factor of 0.014, if not available in the publication

To calculate ECV_CT_ using single-energy protocols, first a non-enhanced scan is acquired, followed by CT angiography for coronary artery assessment and finally, a delayed phase acquisition, most often performed with the same acquisition parameters as the non-enhanced scan [14]. One of the first studies assessing ECV_CT_ used a non-enhanced and a delayed scan 10 min after CM administration for ECV calculation [14]. The scans were acquired with prospective ECG triggering at 120 kV tube voltage and reconstructed with a slice thickness of 3 mm. Several other studies acquired late enhancement scans at different timepoints after CM administration. Hamdy et al. [20] compared ECV measurements obtained with late enhancement scans acquired at 3, 5, and 7 min after CM administration at a tube voltage of 80 kV in patients with known or suspected coronary artery disease. They reported no significant differences of ECV values between the three acquisition timepoints, both for infarcted segments and remote myocardium. However, the delineation of scars on scans with a 5- or 7-min delay achieved higher contrast-to-noise ratio and higher subjective image quality scores compared to the late enhancement scans obtained 3 min after CM administration due to lower tissue enhancement in myocardial segments without scaring [20]. Scully et al. [21] compared ECV at 3 and 5 min in 104 patients before transcatheter aortic valve replacement. They found a small average difference of 0.68% between the two delays, mainly caused by ECV values above 35% (amyloidosis, infarcts).

Other studies on patients with cardiac amyloidosis and in patients with severe aortic stenosis prior to transcatheter aortic valve replacement used a late enhancement scan acquisition at 7 min after CM administration with 120 kV at a 2 mm slice thickness [26], with a 5-min delay at 120 kV and a slice thickness of 1.2 mm [27], or with a 5-min delay using 120 kV for the non-enhanced scan and 80 kV for the late enhancement scan [28].

For dual-energy-based ECV_CT_ calculation, the timing of late enhancement acquisitions varied between studies as well. For dual source dual-energy CT, the acquisition of late enhancement scans ranged between 5 and 12 min after CM administration [18, 24, 29, 30], both with prospective and retrospective ECG-triggering, using energy levels at 80 or 90 kV and 140 or 150 kV [18, 29]. Otha et al. calculated ECV_CT_ using the rapid kV switching technique (80–140 kV) with a late enhancement acquisition performed 7–8 min after CM injection [23]. For dual-layer dual-energy ECV-calculation, the late enhancement scan was acquired 7 min after CM injection, using a tube voltage of 120 kV [22]. ECV quantification with a dual-source photon-counting detector CT has been reported at 5 min acquired at 120 kV, showing a high correlation with single-energy-based ECV measurements [25].

As a conclusion, a late enhancement scan acquisition 3 min after CM injection may be good for quantification of ECV in patients with normal myocardium or diffuse expansion of the interstitium with higher myocardial tissue contrast compared to late enhancement scans at later timepoints after image acquisition. Late enhancement scan acquisition at 5 min may still allow robust ECV_CT_ quantification even with lower tissue contrast of the myocardium but may in addition improve the visualization of focal scars due to lower tissue enhancement of normal myocardium.

### CM injection protocols

Late enhancement scans are acquired as part of a cardiac CT scan to provide additional information of the myocardium. While coronary CT angiography may be acquired with a low dose CM protocol, e.g., using only 30 mL of iobitridol, 350 mg iodine/mL in patients with a body surface area of < 1.7 m^2^ [31], this small amount of contrast media may not be sufficient for ECV calculation due to the low contrast-to-noise ratio of late enhancement scans, mainly depending on the timing of the late enhancement scan and the given total amount of CM [32]. Therefore, the contrast media protocol must be adapted when the acquisition of a late enhancement is considered.

The amount and strategy of CM administration for CT protocols including a late enhancement acquisition vary between studies: Initially, Bandula et al. applied a CM protocol consisting in an injection of a iohexol bolus (1 mg/kg at a rate of 3 mL/s) immediately followed by an infusion of 1.88 mL CM/kg/h (with a maximum of 200 mL) in order to reach an equilibrium between blood and tissue CM concentration, acquiring the delayed phase 25 min after the first bolus injection [12]. While this CM injection scheme may result in a true steady state of CM distribution between the vascular and extracellular space, it is difficult to apply in clinical practice. More practical CM protocols are reported by Hammer et al. [26] using a fixed bolus of 50–60 mL of iopromide 370, by Scully et al. [21] using a fixed bolus of 90 mL iohexol 300 or by Qi et al. [24] using a bolus of 50 mL iopromide 370 for coronary angiography followed by an additional bolus of 50 mL of iopromide 370 before late enhancement scan acquisition. Other groups adapted the CM volume to body weight reaching a total volume of 1.4–1.8 mL/kg of iopamidol [18, 22, 23] or 1 mL/kg of iohexol [27].

Overall, late enhancement scans benefit from a higher volume (in g) of administered contrast media, as a sufficient contrast-to-noise ratio is critical for ECV calculation. Based on the current literature, fixed volumes of 50–100 mL or weight-dependent volumes of 1.4–1.8 mL/kg of contrast media seem to provide adequate image quality for ECV calculation.

### Radiation dose

The additional information provided by ECV_CT_ comes at the expense of an additional CT scan requiring a longer CT slot and at the expense of additional radiation dose.

Comparing the radiation dose reported in literature for CT protocols including a delayed phase is a challenging task, as authors used different CT scanners, tube voltages, and acquisition protocols [10, 12, 14, 15, 17, 18, 20, 22–25]. Nacif et al. reported a mean effective radiation dose of 1.98 ± 0.16 mSv for the late enhancement scan assessed with a single-energy approach [14]. With dual-energy CT, the delayed phase acquisition reached an effective radiation dose ranging from 1.89 mSv to 4.8 mSv [17, 22, 23]. ECV_CT_ assessment with the novel PCD scanner allowed for a low effective radiation dose achieved for the late enhancement scan: 1.2 mSv [0.97–1.75]), according to Mergen et al. [25], and 2.07 ± 1.9 mSv, according to Aquino et al. [33].

In comparison, typical radiation doses of coronary CT angiography range between 1 and 15 mSv [14, 20, 23], whereas more modern CT scanners require lower doses.

## Clinical validation of ECVCT against ECVMRI, histopathology and clinical data

Accurate ECV_CT_ quantification in comparison with endomyocardial biopsy (EMB), which represents the gold standard for the evaluation of myocardial fibrosis, and in comparison with ECV_MRI_, which is considered the clinical imaging alternative to EMB, is imperative to use ECV_CT_ in clinical routine. Table 2 presents a summary of studies correlating ECV_CT_ with histopathology and with ECV_MRI_.

**Table 2:** Summary of studies correlating ECV quantification with CT to ECV measurement with MR imaging and histopathology

**CT–MR imaging correlation**													
	**Number of patients**	**Population**	**CT scanner**	**CT contrast media**	**CT timing for late phase**	**MR scanner**	**MR sequence**		**MR contrast media**		**MR timing for late phase**	**ECV measurement**	**Correlation coefficient and *p*-values**
Nacif, 2012 [14]	24	Heart failure	Aquilion One (Toshiba)	125 mL ± 24 mL of iopamidol (370 mgI/mL)	10 min	3 T Verio (Siemens Healthcare)	MOLLI		0.15 mmol/kg gadopentetate dimeglumine		12 min	Anterior and antero-lateral segments	*r* = 0.82, *p* < 0.001
Treibel, 2015 [34]	26	Amyloidosis	Somatom Sensation 64 (Siemens)	1 mL/kg of iodixanol	5 min and 15 min	1.5 T Avanto (Siemens Healthcare)	shMOLLI		Not provided		15 min	Septal	*r*^2^ = 0.85 (5 min) and 0.74 (15 min), *p* < 0.001
Lee, 2016 [18]	23	Non-ischemic cardiomyopathies	Somatom Definition Flash (Siemens)	1.8 mL/kg of iopamidol (370 mgI/mL)	12 min	3 T Magnetom Trio (Siemens Healthcare)	MOLLI		0.2 mmol/kg gadobutrol		15 min	All myocardial segments	ICC = 0.992 (reader 1) ICC = 0.987 (reader 2)
Hayashi, 2022 [35]	20	Pulmonary hypertension	IQon Spectral CT (Philips)	550 mgI/kg of iopamidol	7 min	3 T Ingenia CX (Philips Healthcare)	shMOLLI		0.2 mmol/kg gadobutrol		15 min	Interventricular junctions, septum, RV and LV free wall	*r* = 0.84, *p* < 0.001 (interventricular junctions) *r* = 0.73–0.79, *p* < 0.001 (septum and LV free wall) *r* = 0.26, *p*: 0.263 (RV free wall)
Aquino, 2023 [33]	29	No specific clinical indication	NAEOTOM Alpha (Siemens)	100 mL of iopromide	5 min	1.5 T Magnetom Avanto (Siemens healthcare)	MOLLI		0.1 mmol/kg gadobutrol		10–12 min	Global and mid-ventricular	*r* = 0.91, *p* < .001
Baggiano, 2023 [36]	39	Dilated cardiomyopathy	Revolution CT (GE)	1.5 mL/kg Iomeron (400 mg/mL)	8 min	1.5 T Discovery MR 450 (GE Healthcare)	MOLLI		0.1 mmol/kg gadobutrol		10–15 min	All myocardial segments	*r* = 0.819, 95% (CI: 0.791 to 0.844)
**CT–histopathological correlation**													
	**Number of patients**	**Study population**	**CT scanner**	**CT contrast media**	**CT timing for late phase**	**Biopsy sample site**		**Samples fixing methods**		**EMB collagen volume fraction**		**ECV CT measurement**	**Correlation coefficient and *p*-values**
Bandula, 2013 [12]	23	Severe aortic stenosis	Somatom Sensation 64 (Siemens)	A bolus of 1 mL/kg + Infusion of 1.88 mL/kg/h of iohexol (300 mgI/mL)	25 min	Interventricular septum		10% buffered formalin		18% [5–40%]		Septal	*r* = 0.71, *p* = 0.0007

Animal models with doxorubicin-induced dilated cardiomyopathy using a dual-energy CT technique in adult New Zealand rabbits [32] and using a single-energy CT technique in beagles [37] showed a strong correlation between ECV_CT_ versus collagen volume fraction (CVF) at histology (*r* = 0.925 and *r* = 0.951, respectively; *p* < 0.001 for both) and between ECV_CT_ versus ECV_MRI_ (*r* = 0.888 and *r* = 0.899, respectively; *p* < 0.001 for both). In addition, ECV_CT_ correlated well (*r* = 0.830–0.907, *p* < 0.05) with the serum fibrosis index (hyaluronic acid, laminin, and type-III procollagen) in the study of Zhou et al. [37].

In the presence of severe aortic stenosis, Bandula et al. challenged ECV_CT_ versus EMB and ECV_MRI_ in 23 patients [12]. ECV_CT_ was assessed on late enhancement scans acquired 25 min after injection of a bolus followed by slow maintenance infusion of iodinated CM. ECV_CT_ significantly correlated with histological extracellular fibrosis, measured as CVF on a septal myocardial sample collected during valve replacement surgery (*r* = 0.71, *p* = 0.0007) and with ECV_MRI_ (*r* = 0.73, *p* < 0.0002) [12].

Cardiac amyloidosis has been found to co-exist in up to 16% of patients with severe aortic stenosis [21]. In a study by Scully et al. including 109 patients with severe aortic stenosis, global ECV_CT_ had an AUC of 0.95 and 0.87 to detect grade 2 and higher, or any grade of amyloidosis, respectively, using 99mTc-DPD scintigraphy as the reference [21]. The authors proposed a threshold of 31% as a clinical screening threshold for scintigraphy and other examinations. Treibel et al. [34] compared ECV_CT_ with ECV_MRI_ in 26 patients with a biopsy-proven systemic amyloidosis and 27 patients with aortic stenosis and demonstrated a stronger correlation between ECV_CT_ and ECV_MRI_ when assessed with a late enhancement scan obtained 5 min (*r*^2^ = 0.85) compared to 15 min (*r*^2^ = 0.74) after CM injection. Moreover, they found that ECV_CT_ was associated with increased laboratory markers (including NT-pro-BNP, troponin), a shorter distance achieved in a 6-min walk test, and the bone scintigraphy amyloid burden (*p* < 0.001) [34].

In patients with known or suspected pulmonary hypertension, ECV_CT_ strongly correlated with ECV_MRI_ in the septum and left ventricular free wall (*r* = 0.79–0.73), while there was only a weak correlation for the right ventricular free wall (*r* = 0.26) [35]. In addition, the mean pulmonary artery pressure had a good correlation to the ECV_CT_ in the anterior right ventricular insertion points (*r* = 0.64), the latter being a potential noninvasive surrogate marker of disease severity in pulmonary hypertension.

In patients with nonischemic cardiomyopathy (hypertrophic or dilated cardiomyopathy, amyloidosis or sarcoidosis), Lee H-J et al. [18] showed that ECV_CT_ calculated from dual-energy late enhancement CT scans obtained 12 min after CM injection was significantly higher compared to ECV_CT_ in healthy subjects (*p* < 0.01). In the same study, authors found similar values for ECV_CT_ (34.5% ± 9.0) and ECV_MRI_ (34.2% ± 9.0) [18]. Nacif et al. also observed elevated ECV values in patients with heart failure compared to healthy volunteers and a good correlation between ECV_CT_ and ECV_MRI_ (*r* = 0.82, *p* < 0.001) but also a small bias (+ 3%) towards higher ECV_CT_ compared to ECV_MRI_ [14]. In contrast, Baggiano et al. compared ECV_CT_ with ECV_MRI_ in 39 patients with dilated cardiomyopathy and showed a strong correlation (all segments, *r* = 0.819) between both methods, while ECV_CT_ values were slightly lower (31.8 ± 6.5% vs 33.9 ± 8.0%, respectively, *p* < 0.001) [36]. In general, myocardial ECV_CT_ assessed with dual-energy CT has an excellent reproducibility and good agreement with ECV_MRI_ in patients with nonischemic cardiomyopathy [18] and heart failure [23, 38]. Moreover, ECV_CT_ is positively correlated with the NYHA classification in patients with non-ischemic heart failure with preserved ejection fraction [24]. ECV_CT_ may even serve as an early biomarker of cardiotoxicity in patients with cardiac dysfunction after anthracycline chemotherapy [39, 40] or chest radiation therapy for esophageal cancer [41], as elevated ECV_CT_ were observed in these patients.

Using a first-generation dual-source photon-counting detector CT, Aquino et al. [33] calculated ECV_CT_ in 29 patients (13 with known cardiomyopathy, 4 with prior myocardial infarction) using both the single-energy and dual-energy approach and then compared these values with ECV_MRI_. The authors found a strong correlation between dual- and single-energy-based techniques (*r* = 0.91, *p* < 0.001) with the dual-energy approach yielding the possibility of reducing radiation dose by 40%. In addition, ECV_CT_ measured by dual-energy photon-counting detector CT had a strong correlation with ECV_MRI_ for midventricular and global quantification (*r* = 0.82 and 0.91, both *p* < 0.001), while ECV_CT_ was higher by approximately 2% compared to ECV_MRI_, similar to the results reported by Nacif et al. [14].

**Fig. 3:**
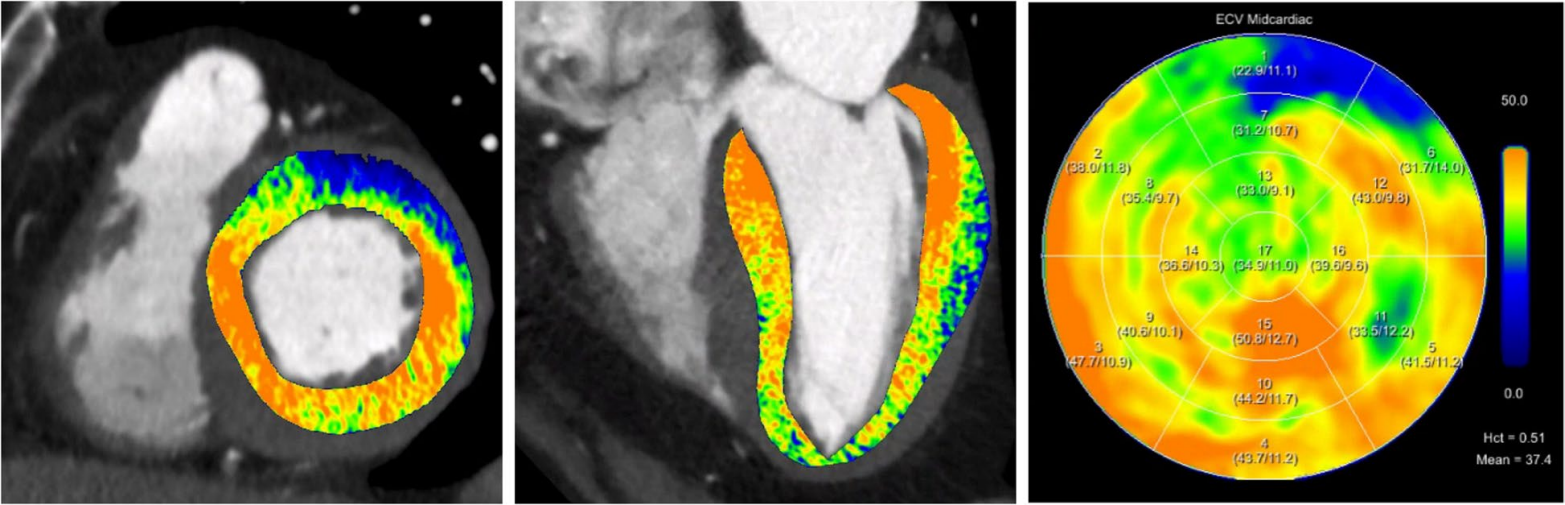
Extracellular volume (ECV) images and maps calculated using the spectral method from late enhancement CT shows elevated myocardial ECV in a 78-year-old male patient with confirmed transthyretin cardiac amyloidosis (ATTR)

**Fig. 4:**
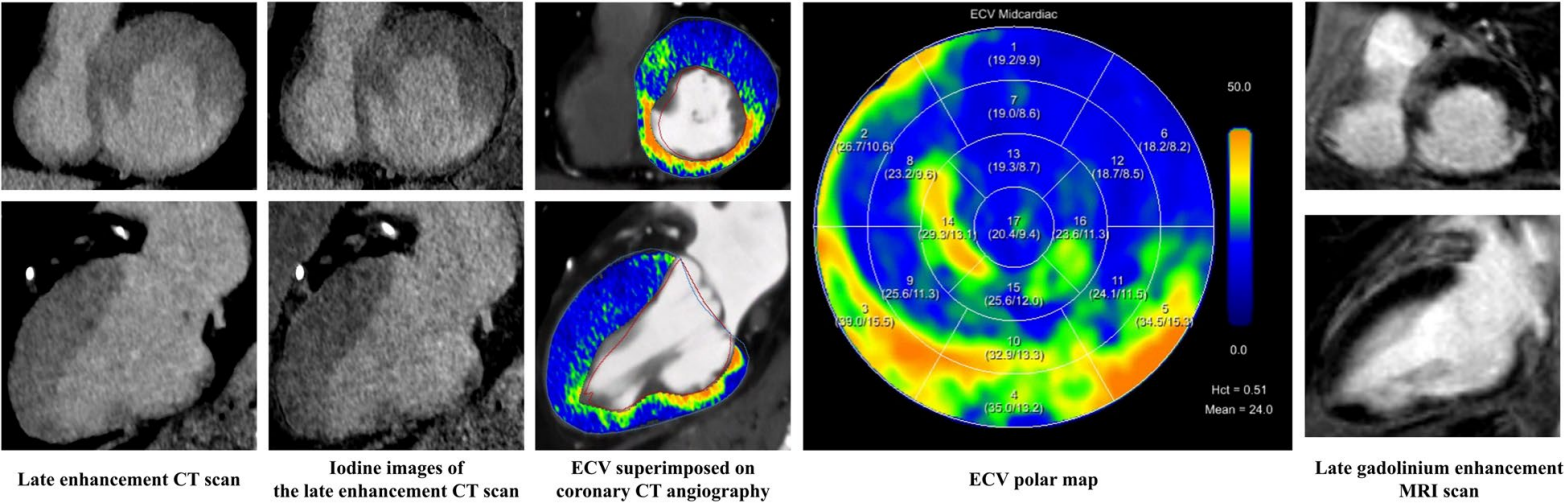
Late enhancement scan and extracellular volume (ECV) calculation in an 89-year-old male patient. Conventional images and iodine maps from the late enhancement scan show increased transmural contrast enhancement of the inferoseptal, inferior and inferobasal myocardium. Elevated ECV_CT_ values were observed in these regions suggesting a focal scar. Findings were confirmed by cardiac MRI. Note the identical extent of the scar on both modalities

Representative image examples of late enhancement scans including ECV_CT_ calculations are shown in Figs. 3 and 4.

## The prognostic value of ECVCT

The prognostic role of ECV_CT_ for predicting outcome has been evaluated in various clinical scenarios and diseases.

ECV_CT_ showed predictive value for outcome of patients undergoing transcatheter aortic valve replacement (TAVR) or surgical valve replacement [10, 21, 26, 28, 29, 42]. Ishiyama et al. demonstrated in 71 patients with severe aortic valve stenosis undergoing TAVR that ECV_CT_ ≤ 32% was associated with a greater reduction in left ventricular mass at follow-up, and ECV_CT_ represented the best independent predictor of hospitalization due to heart failure [28]. Increased ECV_CT_ obtained from pre-procedural TAVR planning CT (ECV_CT_ > 33%) in patients with low-flow low-gradient aortic stenosis was also found to be a significant predictor of heart failure and 2-year mortality after TAVR [10]. Similarly, Scully et al. [21] showed in 112 TAVR patients with lone aortic stenosis (amyloidosis excluded) that medium-term (> 1.5 years) mortality hazard doubled with a 2% increase in ECV. Furthermore, several authors demonstrated that in patients with severe aortic stenosis, ECV_CT_ was significantly correlated with NYHA class, B-natriuretic peptide, echocardiographic left ventricular ejection fraction and E/e’ ratio, and an increased risk of stroke as well as heart failure after transcatheter or surgical valve replacement [26, 29, 43, 44].

In patients with confirmed systemic amyloidosis and variable cardiac involvement, ECV_CT_ correlated with adverse cardiac remodeling and septal ECV_CT_ was independently associated with all-cause mortality in patients with transthyretin (but not light chain) amyloid (hazard ratio: 1.046, *p* < 0.05) [27].

ECV_CT_ was a predictor for major cardiovascular events (MACE, defined as death, ventricular tachycardia or fibrillation and heart failure) at follow-up in patients with dilated cardiomyopathy with an ECV_CT_ of 33% as cut-off to distinguish between patients at high and low risk for MACE [45].

Another potential application of ECV_CT_ is the identification and characterization of myocardial injury in patients with acute chest pain and cardiac troponin elevation. In recent years, coronary CT angiography has gained an increasing role in the emergency setting, thanks to the ability to rapidly rule out obstructive coronary artery disease with simultaneous imaging of the aorta and pulmonary arteries. However, there are acute cardiac conditions with unobstructed coronary arteries, which cannot be diagnosed by coronary CT angiography alone, such as acute myocarditis, myocardial infarction with non-obstructed coronary arteries, and various cardiomyopathies. In this clinical scenario, implementation of a cardiac CT protocol that allows scar detection by late iodine enhancement and ECV_CT_ assessment could be of benefit to establish the proper diagnosis earlier, shorten the hospitalization time, and avoid unnecessary downstream tests (including cardiac MRI), thereby reducing the overall cost to the healthcare system [46]. Thus, Palmisano et al. recently proposed a chest pain protocol in which a late enhancement scan (obtained 10 min after CM application) is performed when there are no findings in coronary CT angiography [47]. In their study, they assessed 84 patients with acute chest pain, 42 of whom had an unremarkable coronary CT angiography. The combination of the arterial phase with late enhancement allowed for the following diagnoses: myocarditis (52%), takotsubo cardiomyopathy (10%), amyloidosis (7%), myocardial infarction with non-obstructed coronary arteries (7%), dilated cardiomyopathy (5%), whereas in 19% of the patients, there were no or inconclusive findings. Therefore, late enhancement cardiac CT may even be helpful in the acute setting and expands the diagnostic yield of the modality.

## Future outlook on potential applications

The continuously increasing availability of dual-energy-capable CT scanners and the recent advent of clinical photon-counting detector CT systems are advantageous for the more widespread use of ECV calculations with CT. The availability of spectral data for ECV calculation render the need for a non-enhanced CT for this purpose unnecessary. A single, spectrally acquired late enhancement acquisition enables both the calculation of the ECV_CT_ and the quantification of cardiac calcifications using virtual non-enhanced images [48]. Thus, future protocols may indicate a shift from a non-enhanced to a standard late phase acquisition at comparable radiation dose but with a higher diagnostic and prognostic value [48]. This approach may be particularly promising in patients planned to undergo TAVR. In these patients, cardiac amyloidosis is a frequent finding [21], and ECV_CT_ is a predictor of long-term prognosis [27].

To non-invasively calculate ECV, the patient’s serum Ht must be measured via blood sampling. However, the Ht may not be always available when cardiac CT scans are performed, particularly in the outpatient setting. In this regard, as shown with ECV_MRI_ [49, 50], some studies proposed to calculate a synthetic Ht by measurements of the attenuation (in HU) of the blood pool of non-enhanced scans or on virtual non-enhanced images from contrast-enhanced dual-energy CT data [51, 52].

Due to the spectral dependency of iron, the calibration curve has to be determined for each CT scanner separately. Moreover, several technical issues must be considered, including the influence of scan parameters such as tube voltage and iterative reconstruction algorithms on tissue or vascular attenuation. In dual-energy CT acquisitions, the monoenergetic reconstruction level may also have an influence on the formula to non-invasively and synthetically calculate the Ht.

As a drawback of this method, synthetic ECV calculation has the potential for a relevant misclassification of ECV for the individual patient, Chen et al. showed that even when using scanner-specific models for calculating synthetic ECV_MRI_, the ECV could be under- or overestimated by 4% compared to ECV_MRI_ calculation using the blood Ht level [50]. This drawback may be similar in synthetic ECV_CT_ calculation as Treibel et al. showed that differences on a per patient level were as high as 8% [52].

## Conclusion

Myocardial ECV is related to myocardial fibrosis, amyloid deposition, or edema and is associated with patient outcome. ECV_CT_ calculation represents a relatively easy, fast, and robust technique to non-invasively detect focal or global increases in myocardial ECV, particularly when using dual-energy capable CT scanners. Several studies have already shown the prognostic value of ECV quantification with CT in various cardiac disease. However, the incremental value of ECV_CT_ comes at the expense of the need to adapt the CM protocols, add radiation dose, and extend the time needed to complete the entire cardiac CT scan. The further availability of spectral CT machines including photon-counting detector CT may further enhance the clinical role of this technique in the near future as spectral data may allow for ECV_CT_ calculation with a lower CM volume and lower radiation dose.

## Data Availability

Not applicable.

## Abbreviations

CM: Contrast media
CT: Computed tomography
CVF: Collagen volume fraction
ECM: Extracellular matrix
ECV: Extracellular volume
ECV_CT_: Extracellular volume quantification with cardiac CT
ECV_MRI_: Extracellular volume quantification with cardiac MRI
EMB: Endomyocardial biopsy
HT: Hematocrit
HU: Hounsfield units
MACE: Major adverse cardiovascular events
MRI: Magnetic resonance imaging
NYHA: New York Heart Association
ROI: Region of interest
TAVR: Transcatheter aortic valve replacement
